# Genetic dissection of drought tolerance and recovery potential by quantitative trait locus mapping of a diploid potato population

**DOI:** 10.1007/s11032-012-9728-5

**Published:** 2012-04-10

**Authors:** A. M. Anithakumari, Karaba N. Nataraja, Richard G. F. Visser, C. Gerard van der Linden

**Affiliations:** 1 Graduate School Experimental Plant Sciences, Wageningen UR Plant Breeding, WUR, PO Box 386, 6700 AJ Wageningen, The Netherlands; 2 Department of Crop Physiology, University of Agricultural Sciences, Bangalore, 560065 India

**Keywords:** Chlorophyll fluorescence, Chlorophyll content, Drought, Potato, QTL, Recovery, δ13C

## Abstract

Potato is the third most important staple food crop in terms of consumption, yet it is relatively susceptible to yield loss because of drought. As a first step towards improving drought tolerance in this crop, we set out to identify the genetic basis for drought tolerance in a diploid potato mapping population. Experiments were carried out under greenhouse conditions in two successive years by recording four physiological, seven growth and three yield parameters under stress and recovery treatments. Genotypes showed significant variation for drought and recovery responses. The traits measured had low to moderately high heritabilities (ranging from 22 to 74 %). A total of 47 quantitative trait loci (QTL) were identified, of which 28 were drought-specific, 17 under recovery treatment and two under well-watered conditions. The majority of these growth and yield QTL co-localized with a QTL for maturity on chromosome 5. Four QTL for δ^13^C, three for chlorophyll content and one for chlorophyll fluorescence (*F*_v_/*F*_m_) were found to co-localize with yield and other growth trait QTL identified on other chromosomes. Several multi-year and multi-treatment QTL were detected and QTL × environment interaction was found for δ^13^C. To our knowledge, this is the first comprehensive QTL study on water deficit and recovery potential in potato.

**Electronic supplementary material:**

The online version of this article (doi:10.1007/s11032-012-9728-5) contains supplementary material, which is available to authorized users.

## Introduction

Potato (*Solanum tuberosum*) is the predominant non-cereal food crop in the world and ranks third in total food consumption after rice and wheat. However, this versatile crop is susceptible to drought stress and is often considered to be drought-sensitive (van Loon 1981), mainly due to its shallow root system, with a depth ranging from 0.5 to 1.0 m (Vos and Groenwold 1986). About 85 % of the total root length is concentrated in the upper 0.3 m of soil. Gregory and Simmonds (1992) showed that the potato root system has relatively small root length per unit area and this makes the potato plant a poor conductor of water. In addition, potato extracts less of the available water from the soil compared to other crops (Weisz et al. 1994). Even short periods of water shortage can reduce tuber production and tuber quality (Miller and Martin 1987). The relative inability of potato to withstand drought limits its productive range to areas with adequate rainfall or suitable irrigation.

Several studies have shown that drought has a drastic effect on the morphological and physiological traits of the potato plant, such as leaf size, leaf number, shoot height (Deblonde and Ledent 2001), rate of photosynthesis, tuber number (MacKerron and Jefferies 1986; Haverkort et al. 1991), tuber yield and biomass (Dalla Costa et al. 1997). The effect of drought on tuber yield depends on the aggregate of morpho- physiological processes, such as photosynthesis, leaf area expansion, leaf senescence, partitioning of assimilates, tuber initiation, bulking and tuber growth (van Loon 1981). In addition, the timing and duration of the stress within the growth period are factors affecting potato yield (Jefferies 1995b), as are the climate and soil conditions. Dramatic reduction of yield occurs when stress coincides with irreversible reproductive processes, making the genetic analysis for drought tolerance at the reproductive stage crucially important.

Potato is a highly heterozygous cross-pollinating crop in which many traits show continuous variation. Cultivar-dependent differences in responses to drought have been reported for *S. tuberosum* (Levy 1983; Jefferies and Mackerron 1987). In addition, several wild species of potato growing in its center of origin in South America have been adapted to harsh and water-scarce conditions (Vasquez-Robinet et al. 2008). This indicates that genetic variability exists within potato and its relatives which can be exploited by breeders to improve drought tolerance. Successful breeding requires exact information on effective drought tolerance traits, their heritability, the genotype × environment interaction and, in addition, suitable selection tools for the traits of interest.

Molecular investigation of complex physiological traits and their genetic relationships to agronomic traits has generated much interest. In recent years molecular mapping approaches have been used to dissect agronomically important and physiologically complex traits that are quantitative rather than qualitative. Quantitative trait loci (QTL) for traits like plant height, maturity, crop emergence, tuber size, and quality traits such as after-cooking darkening, regularity of tuber shape, fry colour and yield components have been identified (Bradshaw et al. 2008). However, insight into genetics and genes underlying QTL that are related to drought tolerance is still limited in potato. Locating QTL for drought tolerance mechanisms by the use of controlled greenhouse or growth chamber experiments combined with field evaluations under relevant conditions should allow the merits of different drought tolerance mechanisms to be established.

In this study we made use of a diploid potato mapping population to increase our understanding of potato plant performance under water stress conditions, and to establish the nature of the phenotypic correlation and genetic association of various physiological and morphological traits. Our main objectives were (a) to evaluate physiological and morphological parameters or secondary characters that are correlated with performance and tuber yield under drought stress and subsequent recovery, (b) to determine the heritability of traits under drought and recovery and (c) to identify QTL for these complex traits in the potato genome as a first step towards identifying candidate genes underlying QTL of drought-related traits.

## Materials and methods

### Plant material

A population of close to 250 genotypes was developed from a cross between clones C (USW5337.3) and E (77.2102.37). Clone C is a hybrid between *S. phureja* PI225696.1 and *S.* *tuberosum* dihaploid USW42. Clone E is a cross between clone C and the *S. vernei*–*S. tuberosum* backcross clone VH34211. Population details can be found in Celis-Gamboa (2002).

### Phenotyping

For the drought experiment, a core set of 94 C × E progeny and their parents were selected. A set of 94 genotypes allows for accurate QTL analysis while still making phenotyping manageable for a number of traits within a greenhouse setup. The experiments were conducted in the greenhouse in two successive years (2008 and 2009) during late spring to summer season at Wageningen UR Plant Breeding, Wageningen University and Research Centre, The Netherlands. The weather conditions and stress period are indicated in Electronic Supplementary Material Table S1. The greenhouse temperature was matched as much as possible to the external air temperature. The air circulation inside the greenhouse was regulated through openings in the roof. Tubers were planted in pots (19 cm^2^ diameter, 3 L volume). Eight replications were maintained in a completely randomized design. Each parental line was repeated four times in each replication to monitor position or corner effects. Irrigation was withheld for six replications starting from the stolon initiation stage. Two replications were maintained as controls with optimal irrigation until the end of each experiment. Three replications out of six were subjected to recovery after 3 weeks of stress period. Samples were collected and measurements were taken for several traits as follows:

#### Leaf relative water content (RWC)

The uppermost fully expanded leaf was sampled, fresh weight (*W*_f_) measured as quickly as possible, and placed in de-ionized water and left for 12–24 h at room temperature. Turgid weight (*W*_t_) was then measured, the leaf sample was dried at 85 °C and dry weight (*W*_d_) measured. Leaf RWC was calculated according to the formula RWC (%) = {(*W*_f_–*W*_d_)/(*W*_t_–*W*_d_)} × 100 (Barrs 1968).

#### Wilting

Drought-induced wilting of genotypes was scored visually based on a 0–5 scale, where 0 is no wilting and comparable to well-watered plants, and 5 is wilted completely including topmost leaves.

#### Carbon isotope composition (δ^13^C)

δ^13^C is a measure of the ratio of stable carbon isotopes ^13^C:^12^C, expressed in parts per thousand (per mil, ‰). In C3 plants, δ^13^C signature is used as a reflection of leaf water-use efficiency (WUE, Condon et al. 2004). Fully expanded mature leaf samples were collected 9 days after initiation of stress. Leaves were oven dried at 65 °C. The leaf material was fine powdered using a MM300 Mixer mill (Retsch Inc., Haan Germany) and samples were analyzed using an Isotope Ratio Mass Spectrometer (IRMS), (Mamrutha et al. 2010) at the Department of Crop Physiology, University of Agricultural Sciences, Bangalore, India and CNR- Institute of Agro-environmental and Forest Ecology, Porano (TR), Italy.

#### Chlorophyll fluorescence (CF)

Drought-induced decreases in photosynthesis have been associated with photo-damage of Photosystem II (PSII) reaction centres (He et al. 1995). Chlorophyll fluorescence is widely accepted as an indication of the energetic behaviour of PSII (Krause and Weis 1991). The potential quantum efficiency of PSII (*F*_v_/*F*_m_) can be used as a reliable indicator to evaluate the energetic/metabolic imbalance of photosynthesis and yield performance across genotypes under water-deficit conditions. Chlorophyll fluorescence parameters including initial fluorescence (*F*_0_), maximal fluorescence (*F*_m_), variable fluorescence (*F*_v_) and maximum quantum efficiency of PSII (*F*_v_/*F*_m_) were monitored on uppermost fully opened and expanded mature leaves under both well-watered and drought-stress conditions using an OS-30p handheld chlorophyll fluorometer (Opti-science, Inc. USA), following the manufacturer’s instructions. The dark adaptation period for all the measurements was about 30 min; measurements were taken (in two replications) at 4 day intervals after the beginning of stress and during the recovery period.

#### Chlorophyll content (CC)

In each genotype five leaves were measured (bottom, upper bottom, middle, upper middle and top leaf) using a SPAD-502 chlorophyll meter (Minolta Co., Ltd. Japan), and the mean of these five values was taken. This was repeated in the biological replicate of the same genotype.

#### Plant height

At the end of stress period the plant height was measured in centimetres (cm) from the soil surface up to the uppermost leaf. Stems were held vertically during the measurement. Measurements were taken on three biological replicates of each genotype.

#### Root length

Roots were washed with water to remove all soil particles adhering to the roots and the longest root length was measured.

#### Shoot and root biomass (fresh and dry)

Shoot and root fresh weights were taken immediately after two harvests: one at the end of stress period and another at the end of recovery. Dry weights were taken after complete drying of the plant material in an oven at 105 °C.

#### Stolon, tuber number and weight

The number of stolons was counted for each genotype in three biological replicates. Stolon ends with diameter >1 cm were considered tubers; tubers were counted for each genotype and total fresh weight of tubers per plant was measured.

### Statistical analysis

All statistical analysis was done with software GenStat 11th edition. Broad-sense heritability (*H* ^2^) was computed from simple one-way analysis of variance (ANOVA) according to the formula *H* ^2^ = (σ_G_^2^/σ_G_^2^ + σ_e_^2^/*r*), where σ_G_^2^ = genetic variance, σ_e_^2^ = environmental variance and *r* = number of replications. Relative reduction (RR) of each trait was calculated as RR = (control–drought)/control and expressed in terms of percentage.

### Genetic map

The genetic map of C × E as described in Anithakumari et al. (2010) was extended with 339 markers from a 768 single nucleotide polymorphism Illumina GoldenGate genotyping array. This array is enriched for markers in genes putatively involved in abiotic stress response. The polymorphic markers were first mapped on parental maps using JoinMap 4.0 (Van Ooijen 2006b) and parental maps were integrated for QTL analysis.

### QTL mapping

MapQTL version 5 (Van Ooijen 2006a) software was used to identify QTL for all traits. Firstly, interval mapping was performed to identify the major QTL. For each trait, a 1,000× permutation test was performed to identify the LOD threshold corresponding to a genome-wide false discovery rate of 5 % (*P* < 0.05). Markers with LOD scores exceeding the threshold were used as cofactors in multiple-QTL-model (MQM) mapping procedures. If new QTL were identified, the linked markers were added to the cofactor list and the analysis was repeated. If the LOD value of a marker dropped below the threshold in the new model, it was removed from the cofactor list and the MQM was rerun. This procedure was repeated until the cofactor list became stable. The final LOD scores were determined by Restricted MQM. The 2-LOD support interval was calculated to estimate the position of significant QTL with 95 % confidence. The integrated maps and QTL were drawn using MapChart 2.2 (Voorrips 2002).

## Results

### Effect of water stress on C × E population

The C × E progeny displayed a wide contrast in drought tolerance, with some individuals surviving and recovering completely after 3 weeks of drought and others completely wilted beyond recovery (Fig. S1). The frequency distribution of genotypes for most of the traits evaluated in this study fitted a normal distribution and parents were always in the middle. The progeny displayed extreme performances for all the traits when compared to the parents, indicating transgressive segregation, as exemplified by the frequency distribution of the traits plant height and δ^13^C (Fig. S2). The results revealed that drought affected all the measured traits, although the severity of stress perceived differed, as indicated by trait mean values for the population (Table 1). The drought stress had a drastic effect on tuber number and tuber weight, as indicated by their relative reduction of about 60 and 80 % respectively. Drought had much less of an effect on number of main stems, with relative reductions of 4–10 % in two successive experiments. The root-to-shoot ratio increased under stress, indicating an increased partitioning of biomass towards root as an adaptive mechanism.

**Table 1:** Population mean values of the traits under control and drought treatments, analysis of variance for the traits under stress and well-watered condition and relative reduction and heritabilities of the traits under drought condition

Trait	Year	Control	Drought	*P* values (two-way ANOVA)			Relative reduction (%)	Heritability (%)
				Genotype (G)	Treatment (T)	G × T		
Number of main stems	2008	4.30	4.13	<0.001	NS	NS	4.03	74.7
	2009	3.46	3.12	<0.001	NS	NS	9.83	59.5
Shoot dry weight (g)	2008	21.03	12.34	<0.001	<0.001	<0.001	41.32	70.1
	2009	17.51	10.36	<0.001	<0.001	<0.001	40.83	61.2
Shoot fresh weight (g)	2008	259.30	102.50	<0.001	<0.001	<0.001	60.47	79.8
	2009	270.11	113.20	<0.001	<0.001	<0.001	58.09	56.5
Plant height (cm)	2008	137.30	98.28	<0.001	<0.001	<0.001	28.42	64.0
	2009	137.93	98.88	<0.001	<0.001	NS	28.31	49.3
Tuber number	2008	7.17	3.21	<0.001	<0.001	0.002	55.23	73.2
	2009	2.60	1.04	0.014	0.003	0.021	60.00	70.6
Tuber weight (g)	2008	33.79	5.53	<0.001	<0.001	<0.001	83.65	69.2
	2009	10.16	1.06	<0.001	<0.001	<0.001	89.57	65.1
δ^13^C (‰)	2008	−31.61	−30.28	<0.001	<0.001	0.032	4.21	58.2
	2009	−30.48	−29.78	0.034	<0.001	NS	2.29	22.6
RWC (%)	2008	83.95	58.73	0.004	<0.001	NS	30.04	36.8
Root dry weight (g)	2009	1.36	1.07	<0.001	0.012	NS	21.32	41.5
Root length (cm)	2009	30.62	24.84	<0.001	<0.001	0.011	18.88	45.1
Root:shoot ratio	2009	0.07	0.11	<0.001	<0.001	NS	−43.50	42.3
Number of stolons	2009	5.84	4.09	<0.001	<0.001	NS	29.97	52.3
Dry biomass (g)	2009	18.87	11.43	<0.001	<0.001	<0.001	39.43	60.0

### Genetic variation of traits under water stress and recovery conditions

In Table 1, the analysis of variance showed that there was highly significant variation (*P* < 0.001) among the genotypes for all the traits under stress conditions. There were significant differences between well-watered and water-stress treatments for all the traits except for number of main stems. Genotypic differences were often specific to the stress response, as there was highly significant interaction between treatment and the genotypes for most of the traits. Plant height showed considerable differences between genotypes; however, consistent interaction between genotype and treatment was not noticed. The majority of traits showed moderate to high heritabilities under stress, ranging from 41.5 to 79.8 %.

Drought-tolerant plants seemed either to maintain water status of tissues, tolerate a reduction in tissue water content, or recover more completely after re-watering. The ability of plants to recover completely after stress is crucial for plants to survive and complete their lifecycle with optimal yield. Under the recovery treatment, all traits varied significantly between progeny. In Table S2, two-way ANOVA revealed significant differences between the drought and recovery treatments. Growth and yield parameters revealed significant interaction between treatment and genotype except for number of main stems and root length. After alleviation of stress, heritabilities for all traits were relatively high when compared to those for the drought-treated plants.

### Evaluation of physiological traits under water stress and recovery

#### Relative water content

Relative water content (RWC) is closely related to cell volume; it may more closely reflect the balance between water supply to the leaf and transpiration rate. Drought generally reduced the relative water content of leaves, as reflected in the mean population values (Table 1). Although there was significant variation among the genotypes for RWC under drought, no sizeable interaction between genotypes and the treatment was observed. RWC was reduced by 30 % upon stress induction and showed 37 % heritability under drought.

#### Carbon isotope composition (δ^13^C)

Significant differences in δ^13^C among the C × E progeny upon stress were found in two successive experiments. Significant interactions between genotypes and treatment were observed in the 2008 experiment. However, there was no such interaction in the 2009 experiment. The heritability of δ^13^C was 58.2 and 22.6 % in the 2008 and 2009 trials, respectively. Mean population values over two experiments between well-watered (more negative) and stress conditions (less negative) clearly revealed enrichment of δ^13^C under stress (Table 1).

#### Chlorophyll fluorescence (CF)

**Fig. 1:**
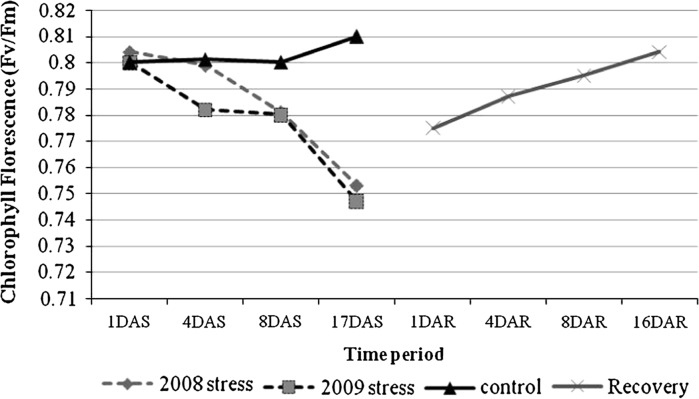
Chlorophyll fluorescence (*F*_v_/*F*_m_) measured during stress and recovery period. *DAS* days after stress, *DAR* days after recovery

The chlorophyll fluorescence measured as *F*_v_/*F*_m_ decreased in the C × E progeny under drought. As expected, under well-watered conditions the mean value of *F*_v_/*F*_m_ was ≥0.8 and the value reduced as the stress period advanced (Fig. 1). There was significant variation among the genotypes under drought and significant differences were observed between treatments. However, significant interaction between genotype and treatment was observed only at the 4th and 16th days after stress initiation. *F*_v_/*F*_m_ had high heritabilities at 1 day after stress initiation but heritabilities decreased as severity of stress increased (Table S3).

After re-watering, plants recovered quickly, as reflected by a marked increase in *F*_v_/*F*_m_ over time, reaching normal values of 0.81 after 17 days of recovery (Fig. 1). Significant variation in *F*_v_/*F*_m_ was observed 4 days after recovery among the progeny tested. We also observed a significant treatment effect over the recovery time period but only at the 4th day was sizeable interaction between genotype and treatment observed. Heritabilities were low under recovery treatment when compared to stress and inconsistent over time (Table S3).

#### Chlorophyll content (CC)

Water stress typically reduces overall plant chlorophyll content and maintenance of chlorophyll stability is considered an important trait. Our results revealed a linear decrease in chlorophyll content with increasing stress severity (Fig. S3). The progeny showed significant variation for chlorophyll content under stress. Except at the 3rd day after stress initiation, there was a significant treatment difference. However, genotype × treatment interaction was not observed. There was a decrease (70–37 %) in heritability of the trait with increasing severity of stress (Table S3).

### Correlations among the measured traits

Under drought conditions δ^13^C showed a highly significant (*P* ≤ 0.001) positive correlation with root dry weight and plant height, and significant positive correlation with number of stolons, shoot dry weight and dry biomass (Table 2), whereas δ^13^C did not have significant correlation to any of the measured traits under well-watered conditions (Table S4). Under well-watered conditions shoot fresh weight, dry weight and root fresh weight showed significant negative correlations with maturity type previously scored in field trials under normal conditions. This implies that genotypes that matured late had higher shoot biomass. However, this correlation was not significant under drought stress (Table 2). Visual wilting symptoms of genotypes showed only a weak correlation of 0.22 and 0.18 with plant height and number of main stems, respectively. Plant height and number of main stems under water limitation conditions had no significant correlation with relative reduction of plant biomass under stress. This indicates that the drought response was only to a limited extent related to growth (size) of the plants and consequent differences in drying down of the pots. This implies that other genetic factors are the main determinants for the response of the plants to drought. Root traits were positively correlated with shoot fresh and dry weight and plant height. Root length was positively correlated with plant height, dry biomass and root-to-shoot ratio and negatively correlated with the number of main stems under drought stress. After recovery, growth parameters were negatively correlated with maturity whereas tuber weight showed a significant positive correlation with maturity type (Table S5).

**Table 2:** Pearson coefficient of correlations among the traits under drought stress at significance levels * *P* ≤ 0.05; ** *P* ≤ 0.01; *** *P* ≤ 0.001

Trait	δ13C	Nr main stems	PM	RDW	R:S wt	SDW	SFW	Tuber wt	Dry biomass	Nr main stems	Nr tubers	Pl ht
Nr stolons	0.400*	–										
PM	−0.003	0.005	–									
RDW	0.473***	0.319	−0.277	–								
R:S wt	0.109	0.057	−0.022	0.247	–							
SDW	0.370*	0.26	−0.337	0.814***	−0.34	–						
SFW	0.264	0.08	−0.307	0.669***	−0.358*	0.873***	–					
Tuber wt	0.187	0.444*	0.012	0.17	−0.077	0.187	−0.078	–				
Dry biomass	0.385*	0.27	−0.337	0.844***	−0.292	0.867***	0.868***	0.188	–			
Nr main stem	0.031	0.144	0.196	−0.314	−0.302	−0.138	0.048	0.1	−0.156	–		
Nr tuber	0.244	0.542***	0.025	0.188	−0.141	0.246	0.047	0.935***	0.244	0.177	–	
Pl ht	0.525***	0.148	0.024	0.518**	0.125	0.353	0.231	0.156	0.373	−0.193	0.157	–
Root length	0.284	−0.048	−0.206	0.628***	0.443**	0.338	0.265	−0.117	0.369*	−0.410*	−0.21	0.395*

Traits: Number of stolons (*Nr stolons*), Plant maturity (*PM*), root dry weight (*RDW*), root-to-shoot dry weight ratio (*R:S wt*), shoot dry weight (*SDW*), shoot fresh weight (*SFW*), shoot to root length ratio (*S:R length*), tuber weight (*Tuber wt*), number of main stem (*Nr main stem*), number of tubers (*Nr tubers*) and plant height (*Pl ht*)

### QTL

**Fig. 2:**
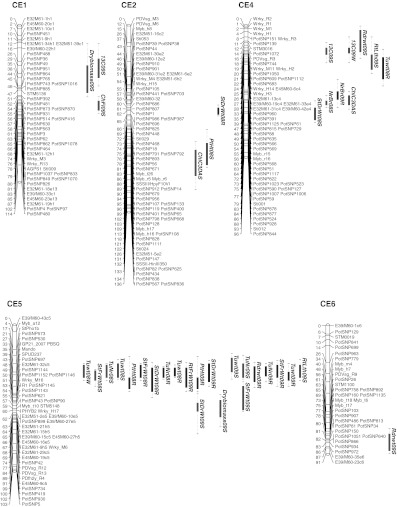

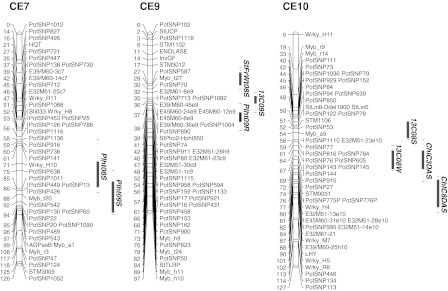
Location of the QTL on the C × E integrated map. Only the linkage groups (chromosomes) with QTL are shown. The *number on the* *left side* is the genetic distance in centiMorgans (cM); marker designations are given on the *right side*. QTL are shown at the *right side* in *vertical bars* with trait names in different *colors* for different treatments (*green*: well-watered; *red*: stress; *blue*: recovery). The *solid vertical bar* shows the 1-LOD interval and the *dotted line* 2-LOD intervals. QTL names are given as trait name followed by year and the treatment, e.g. PlHt08S (Plant height, year 2008, treatment Stress). Abbrevations for QTL names: Number of stem (*NrBr*), Plant height (*PlHt*), Shoot fresh weight (*ShFrWt*), Shoot dry weight (*ShDrWt*), Tuber number (*TuNr*), Tuber weight (*TuWt*), δ^13^C (*13C*), Root fresh weight (*RtFrWt*), Root length (*RtLth*), Chlorophyll fluorescence (*ChFl*), Chlorophyll content (*ChlC*); *DAS* days after stress

QTL analysis was performed in order to identify the genomic regions contributing to the drought-response phenotypes of different physiological, growth and yield traits. The QTL for the measured traits were found on chromosomes 1–10, with no QTL on chromosomes 11 or 12, as shown in Fig. 2. A total of 47 significant QTL were identified on the integrated C × E genetic map under well-watered, drought, and recovery conditions over two successive years. However the number of separate loci may be less, as we found a number of stable QTL over treatments and in two successive experiments for growth and yield parameters, such as number of main stems, plant height, shoot fresh weight, dry weight, tuber number and tuber weight. Table 3 illustrates the flanking markers at one-LOD interval, LOD scores and percentage phenotypic variance explained by each QTL. Two genomic regions on chromosomes 5 and 4 accumulated 31 significant QTL for different traits in stress, well-watered and recovery conditions (Fig. 2). Out of 47 QTL, 28 QTL were detected under stress conditions. Two independent QTL were detected for plant height under stress on chromosome numbers CE7 and CE2 and explained phenotypic variance of 30 and 21 %, respectively. Under recovery, some stable QTL were found for plant height on chromosome CE5 and one on chromosome CE9. The number of main stems had a stable QTL on chromosome CE4 under stress and recovery treatments. Along with other QTL on chromosomes CE4, CE9 and CE2, very stable QTL were found across the treatments for shoot fresh, dry weight, tuber number and tuber weight on chromosome CE5.

**Table 3:** Main characteristics of QTL with a LOD score >4.2 for the traits under well-watered, water-stress and recovery conditions

Name of trait	Year	Treatment	QTL name	Linkage group	LOD score	Interval (cM)	% Variation explained
Number of stem (NrBr)	2008	Stress (S)	NrBr08S	4	4.3	54–64	19.2
	2009	Recovery (R)	NrBr09R	4	5.07	47–55	15
Plant height (PlHt)	2008	Stress	PlHt08S	2	7.05	77–102	21.9
		Stress	PlHt08S	7	6.5	59–71	30.9
	2008	Recovery	Plht08R	5	4.83	34–48	26.2
	2009	Stress	Plht09S	7	6.57	93–98	18.3
	2009	Recovery	Plht09R	9	8.8	38–45	56.7
		Recovery	Plht09R	5	6.09	26–40	6.9
Shoot fresh weight (ShFrWt)	2008	Stress	ShFrWt08S	5	11.34	26–48	42.3
		Stress	ShFrWt08S	9	7.74	16–30	24.6
	2008	Recovery	ShFrWt08R	5	14.37	26–40	57.9
	2009	Stress	ShFrWt09S	5	5.1	47–60	21.6
	2009	Recovery	ShFrWt09R	5	14.95	26–43	60.6
Shoot dry weight (ShDrWt)	2008	Stress	ShDrWt08S	4	4.99	59–71	22.4
	2008	Recovery	ShDrWt08R	5	8.7	35–48	28.8
		Recovery	ShDrWt08R	2	4.98	55–65	10.8
	2009	Stress	ShDrWt09S	5	4.6	54–62	20.2
	2009	Recovery	ShDrWt09R	5	7.89	26–44	35.3
Tuber number (TuNr)	2008	Stress	TuNr08S	5	10.7	26–39	40.8
	2008	Recovery	TuNr08R	5	15.74	26–39	58.3
	2009	Stress	TuNr09S	5	6.05	20–39	25.9
	2009	Recovery	TuNr09R	5	14.6	32–39	51.1
Tuber weight (TuWt)	2008	Well-watered (C)	TuWt08C	5	16.4	26–39	66.7
	2008	Stress	TuWt08S	5	10.48	20–39	40.2
	2008	Recovery	TuWt08R	5	13.19	26–39	40.7
		Recovery	TuWt08R	4	4.96	23–35	9.1
	2009	Stress	TuWt09S	5	7.7	20–33	28.8
	2009	Recovery	TuWt09R	5	15.12	33–39	52.3
δ^13^C	2008	Well-watered	13C08C	10	5.2	63–74	22.8
		Stress	13C08S	10	5.62	47–58	24.3
	2009	Well-watered	13C09C	4	4.8	14–21	20.7
	2009	Stress	13C09S	4	5.1	14–22	18.9
		Stress	13C09S	9	4.55	34–40	12.7
		Stress	13C09S	1	4.1	15–37	15.7
Root fresh weight (RtFrWt)	2009	Recovery	RtFrWt09R	5	6.54	26–48	35.3
Root dry weight (RtDrWt)	2009	Stress	Rdrwt09S	4	5.62	0–18	24.7
		Stress	Rdrwt09S	6	5.35	77–87	17.8
	2009	Recovery	Rdrwt09R	5	8.26	26–43	33.3
Root length (RtLht)	2009	Stress	RtLht09S	4	4.48	25–36	19.2
	2009	Recovery	RtLht09R	5	8.09	26–35	36.3
Chlorophyll fluorescence (ChFl)	2009	Stress	ChFl09S	1	4.57	54–63	19.7
Chlorophyll content (ChlC) 3 Days after stress (DAS)	2009	Stress	ChlC09S	2	7.21	89–102	16.4
		Stress	ChlC3DAS	10	6.95	64–75	15.4
		Stress	ChlC09S	4	5.47	47–58	12.2
Chlorophyll content (ChlC) 8DAS	2009	Stress	ChlC8DAS	10	4.76	82–95	20.8
Dry biomass	2009	Stress	Drybiomass09S	5	6.81	51–62	23.9
		Stress	Drybiomass09S	1	4.5	29–47	15.3

QTL names are given as trait name followed by year and the treatment, e.g. PlHt08S (Plant height, year 2008, treatment Stress)

For the physiological parameter δ^13^C, a total of four QTL were detected, including one on chromosome CE10 under well-watered as well as stress conditions (Fig. 2; Table 3). When the experiment was repeated new QTL were found on chromosomes CE4, CE1 and CE9. Epistasis between QTL for δ^13^C was analysed by two-way ANOVA. Significant interaction (*P* < 0.005) was detected between the QTL on chromosomes CE1 and CE9; the total variance explained by these two chromosomal regions was 28 %. Two independent QTL on chromosome CE10 and CE4 explained 24 and 19 % of variance, respectively. On chromosome CE10, the δ^13^C QTL co-localized with QTL for chlorophyll content, on chromosome CE1 with dry biomass under stress, and on chromosome CE4 with root traits like dry weight, root length under stress and tuber weight under recovery conditions. On chromosome CE9 the δ^13^C QTL was detected in between QTL for shoot fresh weight under stress and plant height under recovery. Under stress, a QTL on linkage group CE1 for chlorophyll fluorescence explained 20 % of the phenotypic variation. Three dependent QTL were detected for chlorophyll content. One major QTL was found on chromosome CE10 for chlorophyll content at different time points during the stress period, along with two other QTL on chromosomes CE2 and CE4.

Root length and root dry weight had QTL co-localizing on chromosome CE4 under stress. Under recovery, however, QTL for root dry and fresh weight and root length were found on chromosome CE5. Two independent QTL were detected for dry biomass under stress on chromosomes CE5 and CE1, explaining 24 and 15 % of phenotypic variation, respectively. Dry biomass QTL co-localized with other traits on chromosome CE5 and with δ^13^C on CE1.

## Discussion

### Genetic variation of parameters under stress and recovery

We screened a diploid potato mapping population for drought tolerance and recovery potential. Most of the drought-tolerance traits are quantitative and difficult to measure in a large number of plants and segregating lines. Indeed the genetic part of the phenotypic variation is often masked by the environmental differences acting on the trait, which in turn makes it difficult to manage the trial and perform relevant measurements in a particular window of time. In the present study we measured a number of growth, physiological and yield parameters in the C × E diploid potato mapping population. There was significant variation for drought response among the progeny, with clear treatment effects and interaction between treatment and genotypes under water-stress and recovery conditions. Progeny showed extreme performances for all the traits when compared to the parents, indicating transgressive segregation. The most plausible cause proposed for transgression is accumulation of complementary alleles at multiple loci inherited from two parents in the progeny (Tanksley 1993). Moderate to high heritability was observed for shoot fresh weight, tuber number, tuber weight, plant height and δ^13^C content under stress and recovery conditions. Heritability estimates provide the basis for selection on the phenotypic performance. Therefore, direct or indirect selection based on these traits may be helpful to improve drought resistance and recovery potential in potato.

### QTL analysis of physiological and growth parameters

The central objective of this study was to identify QTL regions underlying important physiological and growth parameters under water-stress and recovery treatments. We found multi-year as well as multi-treatment QTL for drought response and recovery potential. We identified several QTL for carbon isotope discrimination under well- watered and water-stress conditions. The results indicate that four genomic regions are involved in δ^13^C variation. As would be expected from the complex nature of a trait representing the ratio of two major processes, carbon assimilation (A) and transpiration (T), the control of δ^13^C is strongly polygenic in potato. Any gene(s) that affects either A or stomatal conductance (gs) can have an effect on δ^13^C. No single, large-effect QTL was identified, and QTL breaking the strong correlation between A and T have not yet been discovered in potato. Moreover, temperature and relative humidity have a significant impact on T, influencing QTL detection for δ^13^C. Understanding the inheritance of δ^13^C is crucial for the development of cultivars with high WUE via selection of high δ^13^C lines. Several studies have demonstrated that genetic variation in δ^13^C can be attributed to nuclear factors, and QTL for δ^13^C have been reported in many species including *Arabidopsis* (Juenger et al. 2005; Masle et al. 2005), tomato (Martin et al. 1989), rice (Laza et al. 2006; Takai et al. 2006), soybean (Specht et al. 2001), cotton (Saranga et al. 2004) and barley (Handley et al. 1994; Teulat et al. 2002).

We found a highly significant positive correlation between δ^13^C and plant height under drought. Variation in development and plant height has been shown to affect δ^13^C across different plant species: plant height and flowering date strongly influenced yield-dependent variation in δ^13^C (Laza et al*.* 2006; Ehdaie et al. 1991; Hall et al. 1994; Mckay et al*.* 2003). Phenology and stature also affect plant growth to change biomass and water use under drought, particularly when drought is terminal (Richards et al. 2002). However, the physiological basis for the relationship between δ^13^C and plant height is unclear. In addition, a significant positive correlation was found between δ^13^C and dry biomass of the foliage of the plant, in agreement with studies from Jefferies and Mackerron (1994) and Jefferies (1995a) who found a positive correlation between dry matter production and δ^13^C in two main crop cultivars of potato.

Carbon isotope composition (δ^13^C) as a selection criterion for drought tolerance improvement has been largely documented in cereals, where it has been argued that molecular markers linked to genetic factors controlling δ^13^C could enhance selection in breeding programmes (Condon et al. 2004). The recent release and success of two wheat cultivars with high WUE, namely Rees and Drysdale, for production in rainfed wheat-growing regions of Australia demonstrate that it is effective to breed for water-use-efficient cultivars by selecting for δ^13^C (Richards 2006). The major advantage of using δ^13^C over instantaneous measurements is that sampling is fast and easy with minimal tissue destruction. Sampling can thus be performed in a short time window, which is more preferable for breeding programs. It is also possible to measure gas exchange parameters directly, which would give more detailed information on the assimilation and transpiration. However, these measurements are time-consuming, and not practically applicable in a large population.

Leaf RWC may be used for indirect selection for drought resistance (Chandrasekar et al. 2000). RWC is a measure of plant water status, which also represents variation in water potential, turgor potential and osmotic adjustment. RWC is closely related to cell volume; it may more closely reflect the balance between water supply to the leaf and transpiration rate (Schonfeld et al. 1988). This influences the ability of the plant to recover from the stress and consequently affects yield and yield stability (Lilley and Ludlow 1996). This parameter can also be easily determined, and therefore can be applied for use in large populations. Significant decreases in RWC upon water stress were observed in our study. This result confirms earlier findings in potatoes (Jefferies and Mackerron 1989; Liu et al. 2005). However, no QTL were detected for RWC under well-watered and water-stress conditions.

Several studies have reported on a genotype-dependent decrease of fluorescence quantum yield (*F*_v_/*F*_m_) in potato under drought, and small decreases were associated with drought tolerance, at least in early maturing varieties (Van der Mescht 1999). Ranalli et al. (1997) showed clone-specific variation in CF in drought-exposed potato with a high association with tuber yield. In the current study, drought stress affected CF parameters. The decrease in *F*_v_/*F*_m_ may result from photoinhibition under stress (Baker and Horton 1987). A decline in *F*_0_ could reflect damage to regulatory processes external to P680 (reaction center of PSII), such as impairment of photoprotective processes that facilitate the dissipation of excess energy with the leaf (Angelopoulos et al. 1996). A reduction in *F*_v_/*F*_m_ represents either a reversible photoprotective downregulation or irreversible inactivation of PSII (Baker and Bowyer 1994; Long et al. 1994). Our results revealed significant differences in CF parameters between genotypes and treatments. Although interaction between genotype and treatment was observed in our experiment, the results were not consistent at different time points. Schafleitner et al. (2007) showed that there were no significant differences in CF between potato clones and treatments in a field trial but the same clones in the greenhouse showed significant difference under stress, demonstrating the environmental impact on CF measurements. We found that as the severity of the stress increased, the heritability of *F*_v_/*F*_m_ was decreased. In agreement with Jefferies (1992) and Zrůst et al. (1994), our results also indicated that as severity of the stress increased, *F*_v_/*F*_m_ decreased, as shown in Fig. 1. Hence, CF provides rapid indicators and a method for the study of changes in photosynthetic capacity of potato in response to water stress. In the present drought-response study, an *F*_v_/*F*_m_ QTL was detected on chromosome CE1, which is adjacent to QTL for dry biomass and δ^13^C under stress. Whether there is a causal relationship between these QTL remains to be established.

Chlorophyll is one of the major chloroplast components associated with photosynthesis, and relative chlorophyll content has a positive relationship with photosynthetic rate in barley (Li et al. 2006). Whether a higher CC (i.e. stay green trait) contributes to yield under drought conditions is still under debate (Blum 1998). Many studies in cereals indicate that the stay green trait is associated with improved yield and transpiration efficiency under water-limited conditions (Borrell et al. 2000; Haussmann et al. 2002; Verma et al. 2004). Therefore, maintaining higher CC for a longer period may be one of the strategies for increasing crop production, particularly under water-limited conditions. Saranga et al. (2001) detected different CC QTL under well-watered and dry conditions in cotton. In the C × E population, there was substantial decrease in CC as the severity of water stress increased (Fig. S3). Three independent genomic regions were associated with CC, which may contribute to improved chlorophyll stability under drought. One QTL co-localized on chromosome CE10 with δ^13^C, which also relates to photosynthesis. Other QTL co-localized on chromosome CE2 with plant height under stress and on chromosome CE4 with number of stems and shoot dry weight under stress, suggesting a relationship of CC with growth parameters of plant.

### Association of QTL of different traits

Tuber yield cannot be assessed properly in the early stages of plant growth within the selection processes of a breeding programme. However, physiological and agronomical or morphological parameters measured in the drought-stressed plants may affect tuber formation and bulking at a later stage, and may thus be related to tuber yield performance. Tuber yield was determined by total tuber weight per plant. In our study plant dry matter content, i.e. dry biomass, was highly correlated with root and shoot fresh weights, root length and δ^13^C under stress conditions. Genetic factors which control plant biomass (shoot, root fresh and dry weights) and δ^13^C may affect tuber weight and therefore tuber yield, which would be reflected by localization of QTL for these traits in the same genomic regions.

Time to plant maturity and tuberization are related physiological traits, which are controlled by genetic factors as well as day length. In the present study, time to plant maturity and tuberization were also significantly correlated with shoot and root dry matter content, but only under well-watered conditions and after alleviation of stress. Visual wilting symptoms of genotypes showed only a weak correlation with plant height and number of main stems as a measure of canopy. Plant height and number of main stems under water stress conditions had no significant correlation with relative reduction of plant biomass under stress. This indicates that the drought response was only to a limited extent related to growth (size) of plant and consequent differences in drying down of the pots. This implies that other genetic factors are the main determinants for the response to drought of the plants.

Many researchers (Berg et al. 1996; Schäfer-Pregl et al. 1998; Šimko et al. 1999) identified major QTL for plant maturity on linkage group V in independent mapping populations. On chromosome CE5, we also found stable QTL for plant biomass, dry matter content under stress, plant height, tuber number and tuber weight under recovery. The genetic effects were mostly stable over years in a greenhouse environment. These findings suggest that gene(s) with pleiotropic effects on plant growth, tuberization, plant maturity and tuber yield are located on potato chromosome 5.

On chromosome 10, a QTL of δ^13^C co-localized with a QTL for CC under stress, as assessed with SPAD meter readings under stress. SPAD meter reading is a good indicator of chlorophyll stability, leaf nitrogen and RuBisCo content. These parameters help to evaluate photosynthetic processes, which in turn have possible effects on inter-cellular CO_2_ concentration (*C*_i_) and δ^13^C. Several QTL for δ^13^C overlap with the QTL for other physiological traits and or for yield components.

QTL for root dry weight or root dry mass and root length were co-localized with independent QTL for δ^13^C under water stress on chromosome CE4, together with a QTL for tuber weight under recovery condition. This co-localization of QTL is in agreement with previous research showing that tuber yield, reduction of stomatal conductance, photosynthesis and leaf area significantly correlate with root dry mass under water-deficit conditions (Iwama 2008; Lahlou and Ledent 2005). Consequently, root mass or traits associated with root dry mass could be used as a selection criterion for enhancing tolerance of potato to drought (Iwama 2008; Lahlou and Ledent 2005). The measurement of the root system is tedious and destructive, and thus focussing on shoot or other physiological traits highly correlated with root traits and their QTL co-localization may be another possible method of assessing root traits indirectly. We found that root dry mass and root length highly correlated with plant height and shoot fresh and dry masses under water-deficit and recovery conditions. Hence, the co-localization of QTL for root dry mass and root length on chromosomes CE4 and CE5 with other physiological and growth traits may be of further interest for indirect selection criterion for root traits.

In this study, all these associated traits and their co-localized regions are of interest in terms of plant breeding as they control both important drought-adaptive traits and yield components. Confirmation of the influence of these genomic regions by refining the map or observing similar effects in different populations could help to elucidate biological processes underlying complex traits such as yield or yield stability.

### QTL × E interaction

Variations in climatic conditions are expected to have significant influence on δ^13^C values (Merah et al. 2001). This was the case in the present study; in the 2008 trial a δ^13^C QTL was detected on chromosome CE10, while in the 2009 trial three new QTL were detected on chromosomes CE1, CE4 and CE9 and the QTL on chromosome 10 was not detected. The fact that different QTL were identified for δ^13^C in successive years suggests that QTL × environment interactions influenced the expression of the trait. Documented differences in environmental conditions between the 2 years were observed in the greenhouse, particularly in terms of temperature and relative humidity. Whelan et al. (1973) showed increasing discrimination by 1.2 ‰ per °C rise in temperature. The basis of the biochemical discrimination against ^13^C in C3 plants lies with the primary carboxylation enzyme ribulose-1,5-bisphosphate (RuP2) carboxylase. At a fixed ambient CO_2_ concentration, δ^13^C is negatively associated with the intercellular CO_2_ concentration (*C*_i_). At any moment in time, the *C*_i_ is also negatively correlated with leaf transpiration rate (Hall et al. 1994; Farquhar and Richards 1984). Under water limitations, leaf transpiration efficiency is the major determinant of long-term plant WUE. Under drought conditions, a typical response of plants is simultaneous decrease in photosynthesis and transpiration due to altered leaf conductance (Farquhar et al. 1982). If the supply function (leaf conductance) decreases at a faster rate under stress than the demand function (photosynthesis), this effect should be measurable either as an increase in carbon isotope composition or correspondingly as a decrease in carbon isotope discrimination. In the present study an increase in δ values was observed under drought stress. In the 2009 trial, severity of drought was higher, mainly because of higher temperatures and higher relative humidity when compared to the previous year. We speculate that the δ^13^C QTL on chromosome CE10 specific for the 2008 trial mainly represents the demand function (photosynthesis) which in turn co-localized with a QTL for chlorophyll content, which is a good indicator of leaf nitrogen and RuBisCo content. In the 2009 trial, the higher average temperatures and greater vapour pressure deficit of the air may have acted more on stomatal conductance, resulting in higher δ^13^C. The δ^13^C QTL that were detected on chromosomes CE4, CE1, and CE9 may therefore represent supply functions. Previous reports in wheat and rice documented that variation in temperature, vapour pressure, stomatal aperture and leaf conductance were identified as driving variation in δ^13^C and thereby WUE (Condon et al. 1992; Dingkuhn et al. 1991; Kondo et al. 2004). Further studies are needed to understand precisely how temperature, humidity or vapour pressure, light intensity and other environmental factors contribute to expression of WUE at different stages in potato plant development, to dissect the δ^13^C trait in more detail in different components and to confirm whether the QTL identified in this study are stably expressed in other environments.

### QTL and their implications

QTL identified by genetic dissection of complex characters such as drought tolerance can be used in marker-assisted breeding, which may ultimately improve selection efficiency for yield, reduce problems associated with genotype × environment interactions, and facilitate combining different tolerance traits into a single genotype. For any trait to be used as an indirect selection criterion in breeding programs, its measurement should be easy, rapid and non-invasive. Such an indirect measurement should have a high genetic correlation with the trait that is being selected for and it should have a high heritability. In our study, physiological parameters like RWC, chlorophyll content, δ^13^C, and chlorophyll fluorescence provided rapid indicators of drought stress and methods for the study of the response to water stress of potato. These physiological parameters, as well as plant growth and yield parameters, had moderate to high heritabilities and may be of interest to breeders. From our initial QTL studies, the response of a potato plant to water stress appears to be strongly quantitative and controlled by many genetic factors rather than a few loci of large effect. The strong multigenic nature of the traits and the transgressive variation observed in our mapping population suggest that even lines that do not themselves have high trait value for WUE, tuber number or tuber weight might still contribute favorable alleles. Related wild species might similarly have unique alleles that would be valuable for improvement of potato for drought tolerance. This study constitutes the first knowledge of genetic determinism of important physiological and growth parameters under drought stress and recovery potential in potato. To confirm whether the QTL identified in this study are stably expressed in other environments, multiple location field trials are necessary as well as analysis of these traits at variable growth stages in potato. Further efforts at QTL mapping in this population will focus on trait × QTL interactions. Sequence data from individual QTL and flanking regions can be compared to the genome sequence of the heterozygous diploid line RH (RH 89-039-16) and the doubled monoploid DM1-3 516R44 (DM) potato genome sequence by the Potato Genome Sequencing Consortium (PGSC 2011) to determine the putative candidate genes underlying drought and recovery-specific QTL.

## Electronic supplementary material

Below is the link to the electronic supplementary material.

Supplementary material 1 (DOCX 235 kb)
