# Supplementary material for: Genetic dissection of drought tolerance and recovery potential by quantitative trait locus mapping of a diploid potato population
Source: Mol Breed. 2012 Apr 10;30(3):1413–29. doi: 10.1007/s11032-012-9728-5 (PMC3460171; doi:10.1007/s11032-012-9728-5)

**Supplementary material**

**Table S1** Weather data [temperature and relative humidity (RH)] in the greenhouse at time of experimentation in two successive years

| *Year* | *Temperature (°C)* | | *RH (%)* | | *Stress period* | *Recovery* |
| --- | --- | --- | --- | --- | --- | --- |
|  | Minimum | Maximum | Minimum | Maximum | (Days) | (Days) |
| 2008 | 13.1 | 33.1 | 43.2 | 87.0 | 21 | 12 |
| 2009 | 16.5 | 37.9 | 45.7 | 86.8 | 17 | 30 |

**Table S2** Population mean values of the traits recovery treatment, analysis of variance for the traits under stress and recovery condition and relative reduction and broad sense heritabilities of the traits under recovery condition

| **Trait** | **Year** | **Recovery mean** | **Two-way ANOVA (*P* values)** | | | **Relative**  **reduction (%)** | **Heritability**  **(%)** |
| --- | --- | --- | --- | --- | --- | --- | --- |
|  |  |  | **Genotype (G)** | **Treatment (T)** | **G×T** |  |  |
| Number of main stems | 2008 | 3.9 | <0.001 | NS | NS | 3.2 | 65.4 |
|  | 2009 | 2.7 | <0.001 | NS | NS | –7.5 | 47.4 |
| Shoot dry weight (g) | 2008 | 14.2 | <0.001 | <0.001 | NS | 32.6 | 82.1 |
|  | 2009 | 27.4 | <0.001 | <0.001 | <0.001 | 7.1 | 41.1 |
| Shoot fresh weight (g) | 2008 | 145.1 | <0.001 | <0.001 | <0.001 | 44.0 | 85.4 |
|  | 2009 | 298.8 | <0.001 | <0.001 | <0.001 | 14.5 | 64.7 |
| Plant height (cm) | 2008 | 107.0 | <0.001 | <0.001 | 0.018 | 22.1 | 68.3 |
|  | 2009 | 149.6 | <0.001 | <0.001 | NS | 15.5 | 54.7 |
| Tuber number | 2008 | 2.7 | <0.001 | NS | NS | 53.2 | 81.1 |
|  | 2009 | 5.4 | <0.001 | <0.001 | <0.001 | 47.1 | 85.8 |
| Tuber weight (g) | 2008 | 6.4 | <0.001 | 0.019 | 0.011 | 81.1 | 87.9 |
|  | 2009 | 33.2 | <0.001 | <0.001 | <0.001 | 73.6 | 77.7 |
| Root dry weight (g) | 2009 | 1.9 | <0.001 | 0.012 | 0.04 | 16.2 | 64.6 |
| Root length (cm) | 2009 | 33.5 | <0.001 | <0.001 | NS | 18.0 | 50.4 |
| Root:shoot ratio | 2009 | 0.1 | <0.001 | <0.001 | 0.001 | 10.4 | 44.4 |
| Number of stolons | 2009 | 8.1 | <0.001 | <0.001 | 0.002 | 7.8 | 66.1 |
| Dry biomass (g) | 2009 | 29.4 | <0.001 | <0.001 | <0.001 | 7.7 | 41.6 |

**Table S3** Population mean values of the traits chlorophyll fluorescence (*F*_v_/*F*_m_) and chlorophyll content measured at different time points during stress and recovery period, analysis of variance for the traits under stress and recovery condition and relative reduction and broad sense heritabilities of the traits under stress and recovery condition

| **Trait** | **Time** | **Mean values** | **Two-way ANOVA (*P* values)** | | | **Relative**  **reduction (%)** | **Heritability**  **(%)** |
| --- | --- | --- | --- | --- | --- | --- | --- |
|  |  |  | **Genotype (G)** | **Treatment (T)** | **G×T** |  |  |
| Chlorophyll fluorescence (CF) | 1DAS | 0.8 | NS | NS | NS | 0.00 | 96.2 |
|  | 4DAS | 0.782 | < 0.001 | < 0.001 | < 0.001 | 2.25 | 35.2 |
|  | 8DAS | 0.78 | NS | < 0.001 | NS | 2.50 | 32.7 |
|  | 17DAS | 0.747 | < 0.001 | < 0.001 | < 0.001 | 6.63 | 14.8 |
|  | 1DAR | 0.77 | 0.028 | 0.04 | 0.01 | 3.75 | 22.2 |
|  | 4DAR | 0.78 | NS | 0.034 | NS | 2.50 | 33.7 |
|  | 8DAR | 0.79 | NS | 0.04 | NS | 1.25 | 7.0 |
|  | 16DAR | 0.81 | < 0.001 | < 0.001 | NS | –1.25 | 14.1 |
| Chlorophyll content (CC) | 3DAS | 35.31 | <0.001 | NS | NS | –1.03 | 70.8 |
|  | 7DAS | 34.60 | <0.001 | <0.001 | NS | 1.34 | 47.4 |
|  | 13DAS | 33.45 | <0.001 | 0.028 | NS | 4.13 | 48.3 |
|  | 17DAS | 32.31 | 0.006 | <0.001 | NS | 8.47 | 37.2 |

*DAS* days after stress, *DAR* days after recovery

**Table S4** Coefficients of correlation for the traits under well-watered conditions (harvested at the end of stress period)

| Traits | δ^13^C | Nr stolons | PM | RDW | RFW | R:S dry wt | SDW | SFW | Tuber wt | Nr stems | Nr tubers | Pl ht |
| --- | --- | --- | --- | --- | --- | --- | --- | --- | --- | --- | --- | --- |
| Nr stolons | –0.140 |  |  |  |  |  |  |  |  |  |  |  |
| PM | –0.287 | –0.211 | – |  |  |  |  |  |  |  |  |  |
| RDW | 0.268 | 0.486* | –0.370 | – |  |  |  |  |  |  |  |  |
| RFW | 0.138 | 0.453* | –0.515* | 0.762*** | – |  |  |  |  |  |  |  |
| R:S dry wt | 0.149 | 0.364 | –0.07 | 0.723*** | 0.5623** | – |  |  |  |  |  |  |
| SDW | 0.257 | 0.376 | –0.468* | 0.785*** | 0.5579** | 0.1633 | – |  |  |  |  |  |
| SFW | 0.045 | 0.442* | –0.437* | 0.659*** | 0.54** | 0.0818 | 0.8768*** | – |  |  |  |  |
| Tuber wt | 0.339 | 0.505* | –0.031 | 0.236 | 0.2297 | –0.0355 | 0.3849 | 0.3699 | – |  |  |  |
| Nr stems | –0.377 | –0.1042 | 0.228 | –0.4434* | –0.1768 | –0.4216* | –0.3651 | –0.0839 | –0.2174 | – |  |  |
| Nr tubers | 0.265 | 0.543** | 0.085 | 0.2247 | 0.2615 | 0.0175 | 0.303 | 0.2757 | 0.8006*** | –0.1501 | – |  |
| Pl ht | 0.326 | –0.401* | –0.157 | 0.2092 | 0.1146 | 0.1817 | 0.0989 | –0.0693 | –0.2515 | –0.3952* | –0.2358 | – |
| Root length | 0.040 | 0.031 | –0.172 | 0.5384** | 0.275 | 0.32 | 0.5534** | 0.5176** | –0.1087 | –0.3638 | –0.2189 | 0.308 |

Traits were Number of stolons (Nr stolons), Plant maturity (PM), root dry weight (RDW), root to shoot day weight ratio (R:S dry wt), shoot dry weight (SDW), shoot fresh weight (SFW), tuber weight (Tuber wt), number of main stems (Nr stems), number of tubers (Nr tubers) and plant height (Pl ht).

*Significant at *P*≤ 0.05; **Significant at *P*≤ 0.01; ***Significant at *P*≤ 0.001

**Table S5** Pearson coefficients of correlation for the traits after recovery

| Traits | Nr tubers | Nr stolons | PM | RDW | RFW | R:S dry wt | SDW | SFW | Tuber wt | Nr stems | Pl ht |
| --- | --- | --- | --- | --- | --- | --- | --- | --- | --- | --- | --- |
| Nr stolons | 0.279 | – |  |  |  |  |  |  |  |  |  |
| PM | 0.485*** | 0.021 | – |  |  |  |  |  |  |  |  |
| RDW | 0.051 | 0.437*** | –0.354** | – |  |  |  |  |  |  |  |
| RFW | 0.000 | 0.454*** | –0.372** | 0.975*** | – |  |  |  |  |  |  |
| R:S dry wt | 0.176 | 0.443*** | –0.147 | 0.780*** | 0.779*** | – |  |  |  |  |  |
| SDW | –0.099 | 0.247* | –0.378** | 0.731*** | 0.672*** | 0.174 | – |  |  |  |  |
| SFW | –0.190 | 0.297* | –0.496*** | 0.722*** | 0.678*** | 0.288* | 0.863*** | – |  |  |  |
| Tuber wt | 0.774*** | 0.072 | 0.419*** | –0.102 | –0.165 | 0.010 | –0.168 | –0.28*3 | – |  |  |
| Nr stems | 0.030 | 0.572*** | 0.005 | 0.117 | 0.161 | 0.115 | 0.080 | 0.176 | –0.230* | – |  |
| Pl ht | –0.405*** | 0.087 | –0.421*** | 0.306* | 0.326** | 0.132 | 0.321** | 0.444*** | –0.469*** | –0.004 | – |
| Root length | –0.114 | 0.056 | –0.224 | 0.474*** | 0.416*** | 0.313** | 0.489*** | 0.548*** | –0.079 | –0.138 | 0.358** |

Traits were Number of stolons (Nr stolons), Plant maturity (PM), root dry weight (RDW), root to shoot day weight ratio (R:S dry wt), shoot dry weight (SDW), shoot fresh weight (SFW), tuber weight (Tuber wt), number of main stems (Nr stems), number of tubers (Nr tubers) and plant height (Pl ht).

*Significant at *P*≤ 0.05; ** Significant at *P*≤ 0.01; *** Significant at *P*≤ 0.001

**Fig. S1** C×E progeny showing contrasting responses after 21 days of drought period and one day recovery


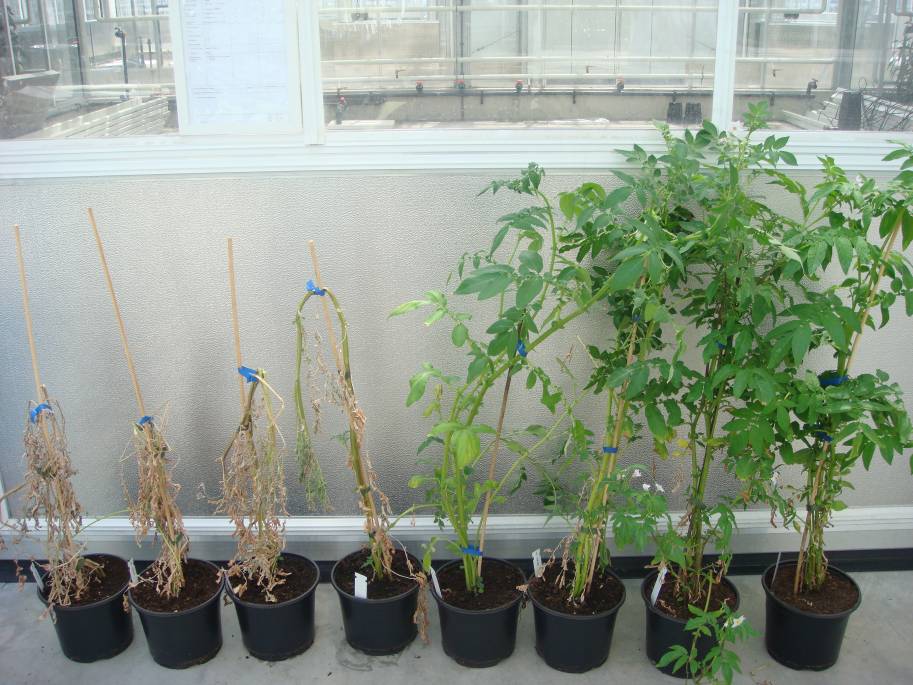


**Fig. S2** Frequency distribution of the traits plant height and δ^13^C measured under drought


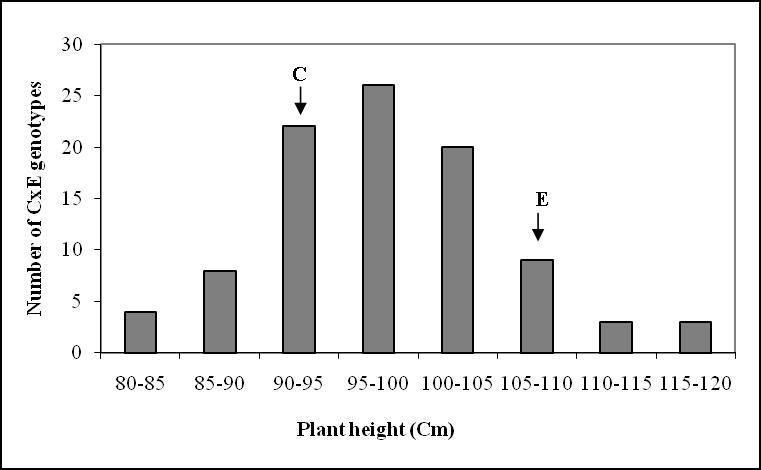


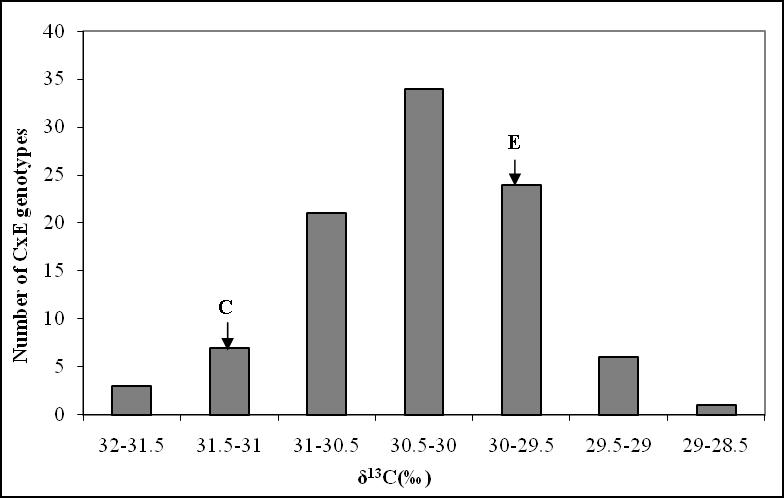


**Fig. S3** Chlorophyll content measured under drought and well-watered conditions at series of time points


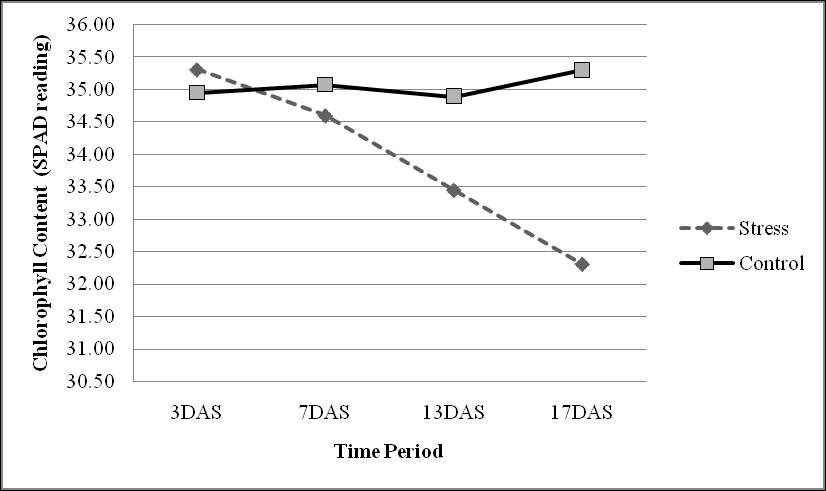

Supplement: Supplementary file 1 — Supplementary material 1 (DOCX 235 kb) [file 11032_2012_9728_MOESM1_ESM.docx]
